# Sputum neutrophils as a biomarker in COPD: findings from the ECLIPSE study

**DOI:** 10.1186/1465-9921-11-77

**Published:** 2010-06-15

**Authors:** Dave Singh, Lisa Edwards, Ruth Tal-Singer, Stephen Rennard

**Affiliations:** 1 University of Manchester, Medicines Evaluation Unit, South Manchester University Hospitals Trust, Southmoor Road, Manchester M23 9QZ, UK; 2 GlaxoSmithKline, Respiratory Medicine Development Centre, Research Triangle Park, NC, USA; 3 GlaxoSmithKline, Respiratory Centre for Excellence in Drug Discovery, King of Prussia, PA, USA; 4 University of Nebraska Medical Center, Omaha, NB, USA

## Abstract

**Introduction:**

The percentage of neutrophils in sputum are increased in COPD patients, and may therefore be a biomarker of airway inflammation. We studied the relationships between sputum neutrophils and FEV_1_, health status, exacerbation rates, systemic inflammation and emphysema, and long term variability at 1 year.

**Methods:**

Sputum samples were obtained from 488 COPD patients within the ECLIPSE cohort. 359 samples were obtained at baseline, and 297 after 1 year. 168 subjects provided samples at both visits. Serum interleukin-6 (IL-6), IL-8, surfactant protein D and C-reactive protein levels were measured by immunoassays. Low-dose CT scans evaluated emphysema.

**Results:**

Sputum neutrophil % increased with GOLD stage. There was a weak association between % sputum neutrophils and FEV_1 _% predicted (univariate r^2 ^= 0.025 and 0.094 at baseline and year 1 respectively, p < 0.05 after multivariate regression). Similar weak but significant associations were observed between neutrophil % and health status measured using the St Georges Respiratory Questionairre. There were no associations between neutrophils and exacerbation rates or emphysema. Associations between sputum neutrophils and systemic biomarkers were non-significant or similarly weak. The mean change over 1 year in neutrophil % was an increase of 3.5%.

**Conclusions:**

Sputum neutrophil measurements in COPD are associated weakly with FEV_1 _% predicted and health status. Sputum neutrophil measurements were dissociated from exacerbation rates, emphysema and systemic inflammation.

## Introduction

Chronic obstructive pulmonary disease (COPD) is a progressive inflammatory airway disease, the most important cause of which is cigarette smoking. COPD is characterised by persistent and progressive airway inflammation [1]. The standard method for classifying disease severity is the measurement of forced expiratory volume in 1 second (FEV_1_) [2]. However, there is a need for biomarkers that are reflective of the inflammatory mechanisms involved in disease pathogenesis [3]. Such biomarkers may be useful for monitoring disease progression, evaluating the effects of therapeutic interventions or identifying disease sub-phenotypes with different clinical characteristics.

A hallmark feature of COPD is the increased numbers of pulmonary neutrophils that can secrete a wide range of pro-inflammatory cytokines and chemokines [1,4,5], as well as proteases that play a role in the development of emphysema. Induced sputum is a non-invasive method that allows evaluation of neutrophil numbers in the airway lumen [6]. The measurement of induced sputum neutrophils fulfils some of the ideal characteristics of a biomarker in COPD; neutrophils are thought to be mechanistically involved in disease pathophysiology [7], can be easily measured in the target organ using a non-invasive method, and are increased in patients with COPD compared to controls [4,5]. There is a need to conduct large cohort studies to further explore the potential utility of this biomarker in COPD patients.

Systemic manifestations such as muscle wasting and cardiovascular disease are common in COPD patients. The relationship between pulmonary and systemic disease is not fully understood. Mechanisms that may cause systemic manifestations include; reduced efficiency of pulmonary gas exchange leading to systemic hypoxia, the systemic absorption of inhaled toxins from cigarette smoke, genetic predisposition to systemic inflammation [8] and a "spill over" of airway inflammation into the systemic circulation [9,10]. If the "spill over" hypothesis is true, one might expect induced sputum neutrophil counts to be associated with systemic measurements of inflammation such as neutrophil numbers in the systemic circulation; a relationship would be suggestive of a "global" activation of neutrophils in COPD patients.

In this analysis we have measured induced sputum neutrophils levels in COPD subjects participating in The Evaluation of COPD Longitudinally to Identify Predictive Surrogate Endpoints (ECLIPSE) cohort [11], with the aim of furthering our understanding of the value of this biomarker in COPD. This paper reports an assessment of the relationships between induced sputum neutrophil counts and FEV_1_, health status, exacerbation rates, systemic inflammation and CT scan quantification of emphysema. Furthermore, we present longitudinal analysis of the change in sputum neutrophil measurements after 1 year to provide an estimate of long term variability.

## Methods

### Subjects

The design of the ECLIPSE cohort study (SCO104960, NCT00292552) has been described elsewhere [11]. Briefly, ECLIPSE is a 3-year multicentre longitudinal prospective study to identify novel endpoints in COPD. Sputum induction was performed in a subset of patients recruited at 14 sites as follows; Lebanon, Denver, Omaha and Hartford (all USA), Halifax, Sainte-Foy, Montreal and Hamilton (all Canada), Bergen (Norway), Edinburgh, Liverpool and Manchester (all United Kingdom), Horn (Netherlands) and Wellington (New Zealand). Inclusion criteria were age 40-75 years, smoking history of > 10 pack-years, a post-bronchodilator ratio between forced expiratory volume in 1 s (FEV_1_) and forced vital capacity (FVC) < 0.7 and FEV_1 _< 80%. Smoking (>10 pack-years) and non-smoking (<1 pack-year) control subjects were enrolled if they were aged 40-75 years and had normal lung function. This study was ethically approved and all participants provided written informed consent.

### Sputum Induction and Processing

The same induction and processing procedure was used at all 14 sites; all site staff received training in these methods. Sputum samples were obtained at the start of the study (baseline) and after 1 year. Sputum induction was performed using 3% saline given as 3 nebulisations each lasting for 7 minutes. Selected sputum was weighed, and samples greater than 0.15 g were mixed with 0.1% DTT on ice in a ratio of 4:1 and processed as previously described to obtain a cell pellet [12]. The cell pellet was re-suspended in cold PBS so that a cell count could be performed using trypan blue to assess the number of viable cells. A cytopsin slide was prepared for differential count. Cytospin preparations were air dried, fixed with methanol and stained with Rapi-diff (Triangle, Skelmersdale, UK). All slides were read independently by two readers, who were blinded to clinical details. Each reader scored 500 cells. This was used to determine the percentage of squamous cells as a measure of sputum quality. Samples with <30% squamous cells were scored as acceptable, 30-60% as fair and >61% as inadequate. After this, additional cells were counted so that a total of 500 non-squamous cells were counted. Agreement for the reads was determined by comparing the differential counts, which had to vary by less than 10% for the cell types averaged. In the event the counts differed, slides were read by a third reader. The results were expressed as a percentage of the total non-squamous count, and a total cell count/ml of sputum.

### Blood biomarker measurements

Whole blood was collected in Vacutainer tubes. Automated neutrophil counts were provided by Quest Diagnostics Clinical Trials (Van Nuys, CA USA). Serum was prepared by centrifugation at 1500 g for 15 minutes. The serum was collected and stored at -80°C until analyzed. Serum concentrations of interleukin-6 (IL-6), and IL-8 were determined by validated multiplexed immunoassays (SearchLight Array Technology, Thermo Fisher Scientific, Rockford, IL, USA). The limits of quantification for IL-6 and IL-8 were 0.4 pg/ml, and 0.8 pg/ml respectively. Serum surfactant protein D (SP-D) was measured using a colorimetric sandwich immunoassay method (BioVendor GmbH, Heidelberg, Germany) according to the manufacturer's instructions. The assay had a validated range of 1.56 to 100 ng/mL. A high sensitivity, sandwich enzyme-linked immunoassay (SearchLight Protein Array Technology, Aushon Biosystems, Inc., Billerica, MA USA) was used to measure CRP. Serum samples were diluted 500- to 10,000-fold for analysis. The lower limit of quantification was 6 ng/ml.

### Exacerbations

Exacerbations were defined as worsening symptoms of COPD and classified as either moderate (requiring treatment with antibiotics or oral corticosteroids) or severe (requiring in-patient hospitalization). At baseline, the patients were asked about the frequency of exacerbations in the previous year. The number of exacerbations during the year after the baseline visit was recorded at clinic visits at 3, 6 and 12 months, and by monthly telephone calls. Sputum samples were not collected within 4 weeks of an exacerbation.

### Health status

Health status was measured using the St Georges Respiratory Questionairre for COPD (SGRQ-C).

### CT Scan

All subjects underwent a low-dose CT scan of the chest at the baseline visit to exclude non-COPD-related disease and to evaluate the degree of emphysema [13]. The CT scans were evaluated at the central imaging unit at the University of British Columbia, Vancouver. Emphysema was assessed by the percentage of the lung with attenuation below -950 HU using the Pulmonary Workstation 2.0 software (VIDA Diagnostics, Iowa City, IA, USA).

### Statistical Analyses

In order to assess the relationship between clinical measurements (pulmonary function, emphysema, and health status) and sputum neutrophils, univariate and multivariate linear regression analyses were conducted. Sputum neutrophils were analysed as percentages and log-transformed counts. The rate of exacerbations over the following year was analysed by negative binomial regression. Robust standard errors for the model coefficients were determined by generalised estimating equations . An offset variable based on the log of the number of days on study was included in the model. Covariates in the regression models included age, gender, body mass index (BMI), concomitant ICS use, smoking history (current or former smoking and pack years), prior exacerbations, and FEV1 % predicted. Spearman correlations were calculated to investigate the association between blood and sputum neutrophils and systemic biomarkers. Bland-Altman plots were constructed to evaluate the repeatability of sputum neutrophil % and neutrophil number/ml over time. To compare the limits of agreement between % and number/ml, the data were log transformed before calculating the limits of agreement. These data were then back-transformed to express the limits of agreements as ratios. SAS^® ^Version 9.1 was used to carry out all analyses. Power curves were generated for change in sputum neutrophil percentage based on a 2 sample t-test with alpha level 0.05 and standard deviation 14.4%.

## Results

### Sputum neutrophils: relationship with pulmonary function

Sputum induction was performed on a total of 538 subjects; 416 subjects at baseline and 346 subjects at year 1. The number of subjects recruited per site varied from 12 to 164 of the 538 subjects. The rate of successful sputum inductions was >50% at every site. Evaluable sputum samples (defined as weight greater than 0.15 g plus sufficient cells to produce cytospin slides) were obtained from 488 subjects, including 168 subjects who produced an evaluable sample at both visits. In total, 359 subjects produced an evaluable sample at baseline, and 297 subjects after 1 year. The demography is shown in table 1; approximately half of the subjects were GOLD stage 2, with the remaining subjects being GOLD stage 3 or 4.

**Table 1:** Demographic characteristics and induced sputum cell counts.

Characteristic	Baseline (n = 359)	Year 1 (n = 297)
Age (y)	63.6 (6.86)	63.4 (6.53)

Gender, Male/Female	225 (63%)/134 (37%)	198 (67%)/99 (33%)

Current/Former Smokers	148 (41%)/211 (59%)	120 (40%)/177 (60%)

Number of pack years smoked	49.2 (28.07)	49.4 (27.97)

Inhaled steroid users	269 (75%)	227 (76%)

Long acting beta-agonist users	279 (78%)	244 (82%)

Long acting anticholinergic users	284 (79%)	243 (82%)

Post bronchodilator FEV1 % predicted	50.2 (15.46)	50.0 (15.94)

Post bronchodilator FEV1 (L)	1.368 (0.49)	1.396 (0.52)

Post bronchodilator FEV1/FVC ratio (%)	44.5 (11.91)	45.8 (11.94)

GOLD Stage II	180 (50%)	154 (52%)

GOLD Stage III	141 (39%)	110 (37%)

GOLD Stage IV	38 (11%)	33 (11%)

Sputum TCC (×10^6/ml)	2.92 (4.92)	3.32 (5.50)

Sputum Neutrophil TCC (×10^6/ml)	2.51 (4.59)	2.89 (5.24)

Sputum Macrophage TCC (×10^6/ml)	0.33 (0.42)	0.35 (0.53)

Sputum Eosinophil TCC (×10^6/ml)	0.028 (0.10)	0.035 (0.13)

Sputum Lymphocyte TCC (×10^6/ml)	0.018 (0.04)	0.015 (0.03)

Sputum Neutrophil %	78.9 (16.4)	82.5 (15.0)

Sputum Macrophage %	16.9 (14.4)	13.9 (13.1)

Sputum Eosinophil %	1.3 (2.6)	1.3 (4.1)

Sputum Lymphocyte %	0.7 (0.8)	0.5 (0.8)
Sputum Epithelial %	2.1 (4.51)	1.7 (3.13)

Data from subjects who produced evaluable sputum samples are shown. Data is mean (SD) or number of subjects (% of subjects) where indicated. Total cell count data was available for n = 293 at baseline and n = 255 at year 1.

**Figure 1:**
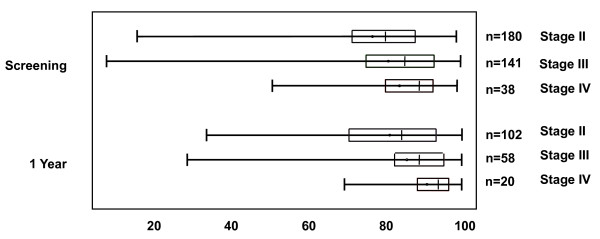
**Sputum neutrophil % shown according to GOLD stage at baseline and year 1**. Medians (lines), interquartile ranges (boxes) and ranges (error bars) are shown.

The mean squamous cell percentages at baseline and year 1 were 11.7% (SD 15.2%) and 12.3% (SD 16.3%) respectively. The sputum cell differential counts expressed as a percentage of the non-squamous cell count for all subjects are shown in table 1. The majority of subjects had a total cell count recorded (293 at baseline and 255 at year 1; due to an error, the total cell count was not recorded for the remaining subjects). The sputum neutrophil % increased numerically with the GOLD staging of disease severity in both the baseline and year 1 samples - see figure 1. This figure shows the wide range of measurements obtained from different subjects. Univariate analysis (tables 2 and 3) showed that the associations between FEV_1 _% predicted and sputum neutrophil % were weak but statistically significant (r2 = 0.025, p = 0.003 and 0.094, p < 0.001 at baseline and year 1 respectively) and remained statistically significant after adjustment by multivariate regression (p = 0.009 and p < 0.001 respectively). Similarly weak, but significant, associations with FEV_1 _were observed for gender (a higher FEV_1 _% predicted was associated with female gender), BMI and ICS use (a higher FEV_1 _% predicted was associated with a higher BMI and no concomitant ICS use). Multivariate analysis showed no association between sputum neutrophil number/ml and FEV_1 _at baseline or year 1 (p = 0.64 and p = 0.19, respectively).

**Table 2:** Linear and multivariate analysis of relationship between post-bronchodilator FEV_1 _% predicted and sputum neutrophil percentage at the baseline visit.

	Linear Regression				Multiple Regression		
**Independent Variables in Model**	**Estimate(SE)**	**p-value**	**R-square**		**Estimate(SE)**	**p-value**	**R-square**

Age	0.095 (0.119)	0.425	0.002		0.197 (0.118)	0.097	0.139

BMI	0.341 (0.141)	0.016	0.016		0.381 (0.140)	0.007	

Concomitant ICS use	5.047 (1.873)	0.007	0.020		6.759 (1.806)	<0.001	

Current smoking status	1.216 (1.658)	0.464	0.002		1.255 (1.689)	0.458	

Pack years	-0.015 (0.029)	0.602	0.001		0.001 (0.029)	0.976	

Gender	7.904 (1.636)	<0.001	0.061		8.785 (1.634)	<0.001	

Sputum neutrophil %	-0.147 (0.049)	0.003	0.025		-0.127 (0.048)	0.009	

Other independent variables were included in this analysis as shown.

**Table 3:** Linear and multivariate analysis of relationship between post-bronchodilator FEV_1 _% predicted and sputum sputum neutrophil percentage at 1 year.

	Linear Regression				Multiple Regression		
**Independent Variables in Model**	**Estimate(SE)**	**p-value**	**R-square**		**Estimate(SE)**	**p-value**	**R-square**

Age	-0.198 (0.137)	0.150	0.007		0.008 (0.129)	0.950	0.209

BMI	0.335 (0.158)	0.034	0.015		0.430 (0.151)	0.005	

Concomitant ICS use	8.241 (2.070)	<0.001	0.051		8.075 (1.960)	<0.001	

Current smoking status	2.486 (1.823)	0.174	0.006		0.959 (1.780)	0.590	

Pack years	-0.056 (0.032)	0.082	0.010		-0.036 (0.030)	0.230	

Gender	7.052 (1.858)	<0.001	0.047		7.588 (1.780)	<0.001	

Sputum neutrophil %	-0.316 (0.057)	<0.001	0.094		-0.272 (0.056)	<0.001	

Other independent variables were included in this analysis as shown.

For the 359 subjects with induced sputum samples at baseline, there was a small decline in FEV_1 _after 1 year of 23.0 mL (p = 0.025). Neither sputum neutrophil percentage nor cell numbers at baseline was associated with the change in FEV_1 _over 1 year (p = 0.71 and 0.33 respectively by multivariate analysis including age, gender, BMI, ICS use, smoking history, number of exacerbations and FEV_1 _% predicted at baseline as independent variables).

### Sputum neutrophils: relationship with emphysema

There was a weak association between sputum neutrophil % and the degree of emphysema as measured by %LAA (r2 = 0.04, p < 0.001 and r2 = 0.09, p = <0.001 respectively at baseline and year 1) by univariate analysis. However, these associations did not persist after adjustment for age, gender, BMI, concomitant ICS use, smoking history, and FEV1 % predicted (p = 0.26 and p = 0.08 at baseline and year 1 respectively).

### Sputum neutrophils: relationship with health status

Univariate analysis (tables 4 and 5) showed a very weak association between sputum neutrophil % and the SGRQ-C score at baseline (r2 = 0.009, p = 0.077). After adjustment, sputum neutrophil % was positively associated with SGRQ-C (p = 0.035). At year 1, this association was significant by univariate linear regression (r2 = 0.022, p = 0.011) but did not reach statistical significance upon adjustment (p = 0.079). Multivariate analysis showed no association between sputum neutrophil count/ml and SGRQ-C at baseline or year 1 (p = 0.1 and p = 0.2, respectively).

**Table 4:** Linear and multivariate analysis of relationship between SGRQ score and sputum neutrophil percentage at the baseline visit.

	Linear Regression				Multiple Regression		
**Independent Variables in Model**	**Estimate(SE)**	**p-value**	**R-square**		**Estimate(SE)**	**p-value**	**R-square**

Age	-0.453 (0.148)	0.002	0.026		-0.471 (0.147)	0.001	0.179

BMI	0.387 (0.176)	0.028	0.014		0.428 (0.174)	0.014	

Concomitant ICS use	-6.813 (2.333)	0.004	0.024		-2.666 (2.293)	0.246	

Current smoking status	-1.941 (2.077)	0.351	0.003		-1.062 (2.089)	0.611	

Pack years	0.051 (0.036)	0.163	0.006		0.086 (0.035)	0.014	

Number of prior exacerbations	2.973 (0.649)	<0.001	0.057		1.923 (0.651)	0.003	

FEV_1 _% predicted	-0.328 (0.065)	<0.001	0.069		-0.307 (0.068)	<0.001	

Gender	1.571 (2.115)	0.458	0.002		4.083 (2.121)	0.055	

Sputum neutrophil %	0.113 (0.063)	0.077	0.009		0.130 (0.061)	0.035	

Other independent variables were included in this analysis as shown. Post-bronchodilator FEV_1 _was used.

**Table 5:** Linear and multivariate analysis of relationship between SGRQ score and sputum neutrophil percentage at year 1.

	Linear Regression				Multiple Regression		
**Independent Variables in Model**	**Estimate(SE)**	**p-value**	**R-square**		**Estimate(SE)**	**p-value**	**R-square**

Age	-0.174 (0.185)	0.348	0.003		-0.282 (0.175)	0.108	0.208

BMI	0.244 (0.213)	0.252	0.004		0.391 (0.207)	0.060	

Concomitant ICS use	-9.516 (2.814)	<0.001	0.038		-3.751 (2.803)	0.182	

Current smoking status	-3.060 (2.454)	0.214	0.005		-1.049 (2.403)	0.663	

Number of exacerbations during year 1	3.936 (0.666)	<0.001	0.107		3.230 (0.683)	<0.001	

Pack years	0.146 (0.042)	<0.001	0.039		0.157 (0.040)	<0.001	

FEV_1 _% predicted	-0.320 (0.076)	<0.001	0.057		-0.190 (0.081)	0.020	

Gender	0.633 (2.565)	0.805	0.000		3.038 (2.490)	0.223	

Sputum neutrophil %	0.205 (0.080)	0.011	0.022		0.138 (0.078)	0.079	

Other independent variables were included in this analysis as shown. Post-bronchodilator FEV_1 _was used.

### Sputum neutrophils: relationship to exacerbations

A total of 496 exacerbations (415 moderate, and 81 severe) were recorded during the 1 year follow up period. Negative binomial regression (tables 6 and 7) showed no relationship between sputum neutrophil % (p = 0.13) or neutrophil number (p = 0.72) at baseline and the number of exacerbations in the following year.

**Table 6:** Negative binomial regression analysis of relationship between exacerbation rates over the one year follow up period and sputum neutrophil percentage at baseline.

	Single Dependent			Multiple Dependents		
**Independent Variables in Model**	**Incidence Rate Ratio**	**95% CI**	**p-value**	**Incidence Rate Ratio**	**95% CI**	**p-value**

Age	0.99	(0.98,1.00)	0.173	0.99	(0.98,1.01)	0.370

BMI	1.00	(0.97,1.03)	0.862	1.00	(0.97,1.02)	0.817

Concomitant ICS use	2.02	(1.47,2.76)	<0.001	1.75	(1.29,2.37)	<0.001

Current smoking status	0.98	(0.76,1.28)	0.903	0.94	(0.73,1.20)	0.605

Pack years	1.00	(0.99,1.00)	0.732	1.00	(1.00,1.01)	0.817

FEV_1 _% predicted	0.98	(0.97,0.99)	<0.001	0.98	(0.97,0.99)	<0.001

Gender	1.17	(0.90,1.52)	0.237	1.38	(1.06,1.81)	0.017

Sputum neutrophil %	1.00	(0.99,1.01)	0.568	0.99	(0.99,1.00)	0.127

Other independent variables were included in this analysis as shown. Post-bronchodilator FEV_1 _was used.

**Table 7:** Negative binomial regression analysis of relationship between exacerbation rates over the one year follow up period and sputum neutrophil number/ml at baseline.

	Single Dependent			Multiple Dependents		
**Independent Variables in Model**	**Incidence Rate Ratio**	**95% CI**	**p-value**	**Incidence Rate Ratio**	**95% CI**	**p-value**

Age	1.00	(0.98,1.01)	0.549	1.00	(0.98,1.02)	0.911

BMI	1.00	(0.96,1.03)	0.815	1.00	(0.97,1.03)	0.825

Concomitant ICS use	2.13	(1.46,3.12)	<0.001	1.77	(1.21,2.58)	0.003

Current smoking status	0.90	(0.67,1.20)	0.470	0.91	(0.69,1.21)	0.520

Log sputum neutrophil number/ml	1.00	(0.95,1.06)	0.951	0.99	(0.94,1.04)	0.724

Pack years	1.00	(0.99,1.00)	0.857	1.00	(0.99,1.01)	0.974

FEV_1 _% predicted	0.98	(0.97,0.99)	<0.001	0.98	(0.97,0.99)	<0.001

Gender	1.18	(0.88,1.57)	0.268	1.46	(1.08,1.98)	0.015

Other independent variables were included in this analysis as shown. Post-bronchodilator FEV_1 _was used.

### Relationship between blood and sputum neutrophils

There was no relationship between blood and sputum neutrophils at baseline, whether expressed as a percentage (r2 = 0.004, p = 0.27) or absolute numbers/ml (r2 = 0.002, p = 0.47). At year 1, there was no relationship between blood and sputum neutrophil percentages (r2 = 0.01, p = 0.076), although a very weak association was observed between blood and sputum neutrophil numbers/ml (r = 0.017, p = 0.044).

### Neutrophils and systemic biomarkers

Table 8 shows the relationships between neutrophil measurements in sputum and blood and systemic biomarkers at baseline. Weak associations were observed between induced sputum neutrophil percentage and serum IL-8 (r2 = 0.02, p = 0.019), and induced sputum neutrophil number/ml and serum SP-D (r2 = 0.02, p = 0.016). Blood neutrophil absolute numbers and percentages were weakly associated with serum IL-6, while neutrophil numbers were weakly associated with serum CRP.

**Table 8:** Univariate associations between serum biomarkers and neutrophil total counts and % in blood and sputum.

	No of subjects	Median (IQR)	Blood neutrophils		Sputum neutrophils	
			
			Total Count	%	Total count/ml	%
C-RP mg/L	134	6.3 (11.0)	r2 = 0.05 ; p = 0.011	NS	NS	r2 = 0.02; p = 0.070

IL-6 pg/ml	331	1.9 (4.3)	r2 = 0.03; p = 0.001	r2 = 0.03; p = 0.001	NS	NS

IL-8 pg/ml	332	7.7 (7.6)	NS	NS	NS	r2 = 0.02; p = 0.019

SP-D ng/ml	279	126.7 (90.6)	NS	NS	r2 = 0.02; p = 0.016	NS

IQR = interquartile range. NS = statistically non-significant

### Longitudinal analysis of induced sputum neutrophil measurements

**Figure 2:**
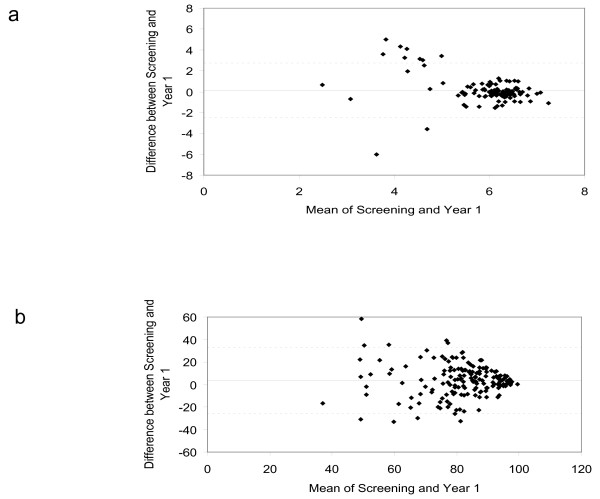
**Bland Altman plots of the mean measurements at baseline and 1 year (x-axis) and the difference between the measurements (year 1 - baseline shown on y-axis) for (a) log10 sputum neutrophil numbers/ml and (b) sputum neutrophil % counts**.

Bland Altman plots for sputum percentage and numbers/ml at baseline and 1 year are shown in Figure 2. For percentages, the mean change was a 3.5% increase at year 1 compared to baseline, with limits of agreement at 32.3% to -25.4%. The changes between repeated measurements at baseline and 1 year were smaller for samples with higher neutrophil %, with most variability observed at lower neutrophil %. The same pattern was observed for neutrophil numbers/ml. Greater variability was observed for neutrophil numbers/ml, as the limits of agreement showed that a repeated measurement can be between 0.003 and 518.7 times the initial measurement. In contrast, for neutrophil %, the ratios lie between 0.61 and 1.50 times the initial measurement.

**Figure 3:**
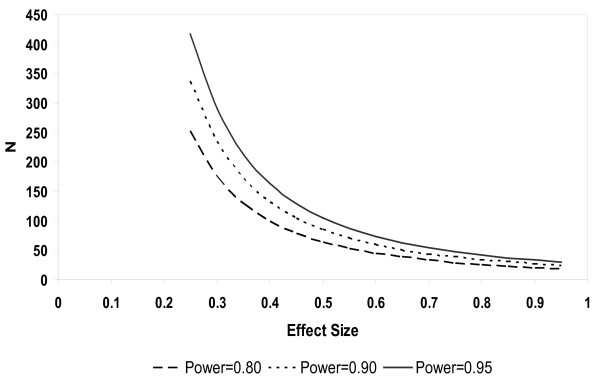
**Power calculations for a reduction in sputum neutrophil % in a parallel group study**. Y axis is the number of subjects required. X axis is the effect size (e.g. 0.9 = 10% reduction).

The within subject standard deviation for sputum neutrophils % was 14.4%. From these data, power curves for future studies with the change in induced sputum neutrophils as an endpoint in an interventional or observational trial in patients with COPD were constructed - see Figure 3.

## Discussion

Neutrophils are thought to play a role in pulmonary inflammation in COPD [7]. Induced sputum neutrophil counts are raised in COPD patients compared to controls [4,5], suggesting that this measurement has potential as a biomarker of airway inflammation in COPD. We have investigated the characteristics of this biomarker in a large group of COPD patients. The wide range of sputum neutrophil measurements was indicative of the degree of between subject variation. Sputum neutrophil measurements were very weakly associated with FEV_1 _% predicted and SGRQ-C scores. Sputum neutrophil measurements did not predict the change in FEV_1 _after 1 year, or the rate of exacerbations, and were not related to the degree of emphysema. Additionally, we found little evidence of any association between sputum neutrophils and biomarkers of inflammation in the systemic circulation, including blood neutrophil counts, CRP and SP-D.

Our findings raise the question; what is the value of sputum neutrophil measurements in COPD ? There is a need for biomarkers of airway inflammation in COPD patients [3]; for example in clinical trials of anti-inflammatory interventions or in longitudinal observational studies of the natural course of the disease. Sputum neutrophil levels are characteristically raised in COPD patients [4,5], but this measurement of airway inflammation is only very weakly associated with FEV_1 _and health status. Our results suggest that measuring sputum neutrophils in COPD patients is principally a tool to assess the burden of airway inflammation; it is not a major surrogate of the other clinical and pathophysiological abnormalities measured in this study.

Generally, any weak but significant associations between clinical parameters and sputum neutrophils were observed for percentages and not numbers/ml. Neutrophil numbers/ml also displayed a high degree of variability over 1 year, and so appear to be less informative than the measurement of neutrophil % in COPD patients.

A previous study in 44 COPD patients showed a statistically significant relationship (p < 0.001) between FEV_1 _% predicted and sputum neutrophil percentage; the r value was reported as -0.54, hence r2 = 0.29 [14]. This is a weak relationship, and the current study in much larger numbers of subjects showed an extremely weak relationship (r2 < 0.1) that again was statistically significant (p < 0.001 at both baseline and year 1). This suggests that sputum neutrophil numbers play only a very minor role as a predictor of the degree of airflow obstruction in COPD patients. Supporting evidence for this observation comes from studies using principal component analysis that have shown induced sputum neutrophil measurements to be dissociated from pulmonary function measurements [15,16]. While it is known that the number of neutrophils in walls of the small airways are related to the severity of airflow obstruction [1], our findings and previous studies indicate that this relationship is very weak for measurements of the number of neutrophils in the airway lumen.

A biomarker that could predict the rate of lung function decline in COPD would be of great clinical usefulness. It has previously been reported in a limited number of COPD patients (n = 45) that the total neutrophil number/gram sputum is related to the subsequent decline in pulmonary function over 7 years, although no analysis for neutrophil % was presented [17]. Additionally, a study in 38 smokers showed that lung function decline over 15 years was associated with sputum neutrophil percentage [18]. It should be noted that the sputum samples were obtained retrospectively at the end of the 15 year period. Consequently, this was not a prospective study evaluating whether sputum neutrophils are a biomarker of subsequent lung function decline. Our study had a much larger number of patients (n = 359), than these previous studies [17,18] but a shorter follow up period (1 year). The decline in FEV_1 _was 23 mls over this follow up period. This is a rate of decline that is less than might be expected in a COPD population and may reflect a Hawthorne effect i.e. the rate of decline in these patients has been reduced simply by inclusion in a clinical study. Additionally, it is likely that a 1 year follow up in this population was insufficient to properly study longitudinal decline. There was no relationship between baseline neutrophil numbers or percentage and the change in FEV_1 _over this time period. The ECLIPSE study will run for at least 3 years [11], and it will be of interest to observe if sputum neutrophil measurements can predict FEV_1 _decline over a longer time period.

Neutrophils are known to be involved in the pathogenesis of emphysema, through the secretion of proteases such as neutrophil elastase [7,19]. Other important factors involved in the pathogenesis of emphysema include protease production by other cell types such as macrophages, and the degree of anti-protease activity [19]. We observed no association using multivariate analysis between sputum neutrophil counts and the degree of emphysema measured by HRCT. This negative finding suggests that the sputum neutrophil number is not reflective of the protease/anti-protease balance, which may not be surprising as the number of neutrophils does not inform us about overall protease and anti-protease levels in the lungs. A previous study in smaller numbers of COPD patients has also reported no association between sputum neutrophils and HRCT quantification of emphysema [20].

It is known that sputum neutrophil numbers are raised in COPD exacerbations [21,22]. We were able to test whether sputum neutrophil measurements during the stable state are predictive of the future rate of exacerbations, but found no evidence to support this hypothesis. It is known that a subset of COPD patients suffer with more frequent exacerbations, which is associated with a faster decline in lung function [23]. It is possible that these frequent exacerbators have increased levels of airway inflammation even during the stable state between exacerbations, but in our study population any such increase was not detectable by measuring sputum neutrophils.

The factors that impact quality of life in COPD are not well understood, and it is possible that the degree of airway inflammation is a contributor. A previous study showed a weak association between sputum macrophage numbers and SGRQ-C, but no relationship to sputum neutrophil numbers [24]. The current study had a larger sample size, but still observed a very weak relationship between SGRQ-C scores and sputum neutrophils. Other weak predictors of SGRQ-C score were the number of previous exacerbations, smoking history and FEV_1 _% predicted. This analysis underscores the multicomponent nature of COPD, with quality of life being determined by a range of different clinical and pathophysiological factors.

It has been proposed that systemic inflammation in COPD is a "spill-over" of inflammation from the lungs [9,10]. Alternatively, systemic and pulmonary inflammation in COPD may arise due to distinct mechanisms. We observed no consistent relationship between sputum and blood neutrophil numbers or percentages. This argues against any common mechanisms controlling neutrophil recruitment into these separate compartments. Similarly, we found no strong relationships between sputum neutrophils and systemic biomarkers of inflammation. It appears that the degree of systemic inflammation in COPD is independent of the level of airway neutrophils.

There are multiple mechanisms by which neutrophils may be recruited into the airways. Thus, it is a reasonable conjecture that similar numbers of neutrophils present in the airways of different COPD patients may reflect different pathophysiological processes. This is consistent with the recognised clinical heterogeneity of COPD.

The mean change in sputum neutrophil percentages over 1 year was only 3.5%, suggesting good reproducibility. However, the limits of agreement, which define the level of variability that can be expected from a repeated measurement in an individual, were approximately 30%. The most variability was observed in samples with a low neutrophil percentage, which suggests that a low neutrophil is often a transient phenomenon, and that repeated measurements "regress to the mean" which is a higher value.

The longitudinal assessment of change at 1 year can be used to design future long term observational studies or therapeutic trials in COPD. Previous studies with repeat sputum measurements have been of shorter duration, usually 3 months or less [25,26]. Our finding that the mean change in sputum neutrophil percentage was 3.5% can be used to guide the natural variability in this measurement that can be expected over 1 year, and this variation appears to be greatest for individual subjects with lower neutrophil percentage counts. The power calculations presented can be used for future clinical trials; for example, to detect a difference of 10 percentage points in mean sputum neutrophil % between two groups with 80% power would require 34 subjects per treatment arm based on a two-sample t-test and alpha level 0.05. As sputum neutrophils appear to be only weakly associated with clinical parameters such as FEV_1_, exacerbation rates and quality of life, it is unclear at present whether reducing sputum neutrophil numbers would actually produce a clinical benefit in COPD patients. The data provided in this paper shows the sample size that is required to be able to show that a novel therapeutic intervention, such as an inhibitor of neutrophil chemotaxis [27], can reduce airway neutrophil numbers in COPD. The possible clinical benefits of this type of approach remain unclear.

A strength of the current study is its size and multicentre design. All studies, including ECLIPSE, that have evaluated induced sputum in COPD to date have recruited "convenience" samples. Thus it is likely that all studies to date have assessed populations that reflect some degree of selection bias. The current study, which recruited a large number of subjects from 14 sites is likely to have recruited a more heterogeneous sample of COPD patients than studies conducted at single centres with smaller numbers of subjects.

In conclusion, sputum neutrophil counts do not appear to be a major surrogate of other clinical or pathophysiologal abnormalities in COPD. The value of this biomarker in COPD appears to be principally as a tool for measuring the burden of neutrophils in the airways.

## Competing interests

DS has received lectures fees, support for conference attendance, advisory board fees and research grants from a range of pharmaceutical companies including GSK, Chiesi Pharmaceuticals, AstraZeneca, CIPLA, Novartis. Forest, MSD, Boehringer and Allmiral

LE and RT are employees of GSK

SR has consulted or participated in advisory boards for: Able Associates, Adelphia Research, Almirall/Prescott, APT Pharma/Britnall, Aradigm, AstraZeneca, Boehringer Ingelheim, Chiesi, CommonHealth, Consult Complete, COPDForum, DataMonitor, Decision Resources, Defined Health, Dey, Dunn Group, Eaton Associates, Equinox, Gerson, GlaxoSmithKline, Infomed, KOL Connection, M. Pankove, MedaCorp, MDRx Financial, Mpex, Novartis, Nycomed, Oriel Therapeutics, Otsuka, Pennside Partners, Pfizer (Varenicline), PharmaVentures, Pharmaxis, Price Waterhouse, Propagate, Pulmatrix, Reckner Associates, Recruiting Resources, Roche, Schlesinger Medical, Scimed, Sudler and Hennessey, TargeGen, Theravance, UBC, Uptake Medical, VantagePoint Management. SR has given lectures for: American Thoracic Society, AstraZeneca, Boehringer Ingelheim, California Allergy Society, Creative Educational Concept, France Foundation, Information TV, Network for Continuing Ed, Novartis, Pfizer, SOMA. SR has received industry-sponsored grants from: AstraZeneca, Biomarck, Centocor, Mpex, Nabi, Novartis, Otsuka.

## Authors' contributions

DS was involved in study design and data interpretation, and drafted the manuscript. LE was the lead for statistical analysis. RT was involved in study design and data interpretation. SR was involved in study design and data interpretation

All authors have read and approved the final manuscript.
